# Percutaneous sigmoid fixation for management of recurrent sigmoid volvulus: a randomized controlled clinical trial

**DOI:** 10.1186/s13017-026-00701-2

**Published:** 2026-05-20

**Authors:** Said Negm, Ahmed Khaled AboZeid, Ahmed Shafiq, Joseph Rizk I. Awad, Abdelrahman Nawar, Pola Nagy A. Wassef, Amr A. Abdelghan, Mohamed Adel Saleh, Mahmoud R. Elballat, Elsayed Elhendawey, Ahmed M. Mahgoub, Abdel-Baset Mohamed, Mohamed S. Bayomi, Alaa A. Faid, Karim M. Fathy, Ahmed Farag

**Affiliations:** Faculty of Medicine, Zagazig University, Zagazig City, Egypt

**Keywords:** Sigmoid volvulus, Percutaneous fixation, Endoscopic management, Anchor device

## Abstract

**Background:**

Sigmoid volvulus stands out as a frequent surgical emergency, particularly in older adults. Initial management centers on prompt detorsion of the affected colon segment, coupled with strategies to avoid future episodes. Preventing recurrence remains a persistent clinical challenge. Advances in endoscopic technology have positioned this modality as a pivotal tool in volvulus care, offering the dual advantage of therapeutic detorsion and diagnostic evaluation of predisposing factors.

**Methods:**

We prospectively included all patients who presented to the emergency department at Zagazig University Hospital with recurrent sigmoid volvulus from August 2024 to August 2025. Sixty-six participants were randomized equally (n = 33 per group) to percutaneous endoscopic fixation or surgical intervention (control group: open or laparoscopic sigmoidopexy or sigmoidectomy under general anesthesia) using sealed envelopes with third-party-generated random assignments. The protocol received prospective institutional review board approval from Zagazig University Faculty of Medicine (IRB #633/1-Sep-2024) and was registered retrospectively on ClinicalTrials.gov in September 2025 (NCT07155304). The trial was conducted under the code of ethics of the World Medical Association (Declaration of Helsinki) for studies involving human subjects. And all participants provided written informed consent. Reporting followed CONSORT guidelines.

**Results:**

Procedure-related complications included small bowel injury, sigmoid perforation, skin necrosis, pulmonary infection, intra-abdominal abscess, peritonitis, fecal fistula, recurrence, and mortality. In the percutaneous endoscopic fixation group, rates were 1 (3%) for small bowel injury, 1 (3%) for sigmoid perforation, 2 (6%) for skin necrosis, 3 (9%) for pulmonary infection, 3 (9%) for abscess, 3 (9%) for peritonitis, 0 (0%) for fistula, 1 (3%) for recurrence, and 0 (0%) for mortality. Corresponding figures in the surgical cohort group 2 (6%), 0 (0%), 4 (12%), 5 (15%), 4 (12%), 4 (12%), 4 (12%), 1 (3%), and 3 (9%). Quality-of-life assessments categorized outcomes as excellent, good, or poor: endoscopic group, 24 (73%), 5 (15%), 4 (12%); surgical group, 17 (52%), 11 (33%), 5 (15%).

**Conclusion:**

Percutaneous endoscopic fixation provides a lower-morbidity and mortality alternative approach for patients with recurrent sigmoid volvulus without the need for general anesthesia.

## Introduction

Sigmoid volvulus constitutes a common acute surgical condition, disproportionately affecting elderly individuals [1]. “Recurrent sigmoid volvulus means the colon has twisted at least twice after an earlier de-torsion (by tube or scope) [1]. It almost always happens in older people who have a very long, floppy sigmoid loop plus ongoing constipation, dementia, Parkinson’s, psychiatric illness or heavy opioid use [2]. Without some kind of fixation, recurrence becomes frequent with increased morbidity and mortality [2]. Therefore, we wanted to test a less invasive endoscopic method to fix it” [3]. Its presentation spans a spectrum from incidental discovery to life-threatening peritonitis secondary to colonic ischemia and necrosis, underscoring the urgency of intervention [2]. Standard care prioritizes emergent detorsion to restore colonic patency, followed by measures to mitigate recurrence [3]. Noninvasive attempts begin with rigid sigmoidoscopy or rectal tube decompression, suitable only in the absence of ischemic features [4]. When these fail, flexible colonoscopy offers a reliable alternative for detorsion [5]. In contrast, established ischemia mandates emergent resection, typically via Hartmann’s procedure with proximal colostomy and delayed anastomosis [6].

Recurrence prophylaxis following successful detorsion—whether by tube or endoscopy—poses ongoing difficulties [7]. Traditional strategies involve sigmoid resection or mesofixation, performed openly or laparoscopically; however, these carry substantial morbidity and mortality, amplified in comorbid geriatric populations [8]. Endoscopic techniques have evolved to address these limitations, providing both therapeutic detorsion and diagnostic insights: mucosal viability assessment, precise localization of the torsion point (often manifesting as a spiral mucosal fold), and exclusion of alternative obstructive etiologies. Acute detorsion succeeds in 75–95% of cases endoscopically, though recurrence approaches 90% without adjunctive fixation [9, 10]. Herein, we explore percutaneous endoscopic sigmoidopexy using anchor devices to secure the colon to the anterior abdominal wall, aiming to curtail recurrence while minimizing perioperative risks in vulnerable patients.

### Objectives

This trial evaluated the efficacy, safety profile, and clinical benefits of percutaneous endoscopic fixation for recurrent sigmoid volvulus, with particular emphasis on high-risk surgical candidates.

### Patients and methods

#### Participants

Consecutive adults presenting with recurrent sigmoid volvulus to the emergency department at Zagazig University Hospital between August 2024 and August 2025 were eligible. Protocol approval was obtained prospectively from the Zagazig University Faculty of Medicine Institutional Review Board (IRB #633/1-Sep-2024), with retrospective registration on ClinicalTrials.gov (NCT07155304, September 2025). The trial was conducted under the code of ethics of the World Medical Association (Declaration of Helsinki) for studies involving human subjects., and all participants provided written informed consent. Reporting followed CONSORT guidelines.

“Patients were included if they had recurrent sigmoid volvulus (at least two documented episodes after previous successful de-torsion) and were considered high-risk for conventional open or laparoscopic surgery because of age over 60, heavy comorbidity load (severe heart or lung disease, chronic liver or kidney problems, previous strokes, or poor nutrition) or chronic constipation. ASA class I–IV was simply recorded to describe overall health; it was not the deciding factor for entry. We still excluded ASA V–VI, clear ischemia on imaging or scope, previous lower abdominal operations, and BMI above 35.” Sample size estimation targeted mortality differences as the primary outcome, informed by prior literature indicating 16–42% post-surgical versus 9.7% post-percutaneous rates.[11] Using an 80% power, α=0.05, uncorrected chi-square test, and 20% attrition allowance, we calculated 33 patients per group. We randomized 1:1 using sealed opaque envelopes numbered in sequence. A statistician who had nothing to do with patient care generated the random list on a computer. The envelopes were opened by a research coordinator who was blinded to the sequence only after the patient was confirmed eligible and had given written consent.” There were no extra rules for putting a patient in the percutaneous endoscopic arm as the randomization decided everything after the initial de-torsion was done

#### Diagnostic evaluation

History and exam: sudden massive bloating, pain, vomiting, no flatus/stool, and known previous volvulus episodes. B- Plain erect abdominal film: looking for the typical coffee-bean or kidney-bean dilated loop high on the left. C- Contrast CT abdomen/pelvis: whirl sign at the sigmoid, clear transition point, and importantly no signs of ischemic/gangrenous bowel (thick wall, pneumatosis, portal gas, free fluid/air) d- Urgent flexible endoscopy: sigmoidoscopy or colonoscopy to see the spiral twist, check mucosa for viability, and usually de-torsion at the same time if the bowel looks pink.

All patients needed at least two of these (usually CT plus scope) to be sure before we enrolled them. This matches what most guidelines say (Vogel 2016 and others).

#### Interventions

**Fig. 1:**
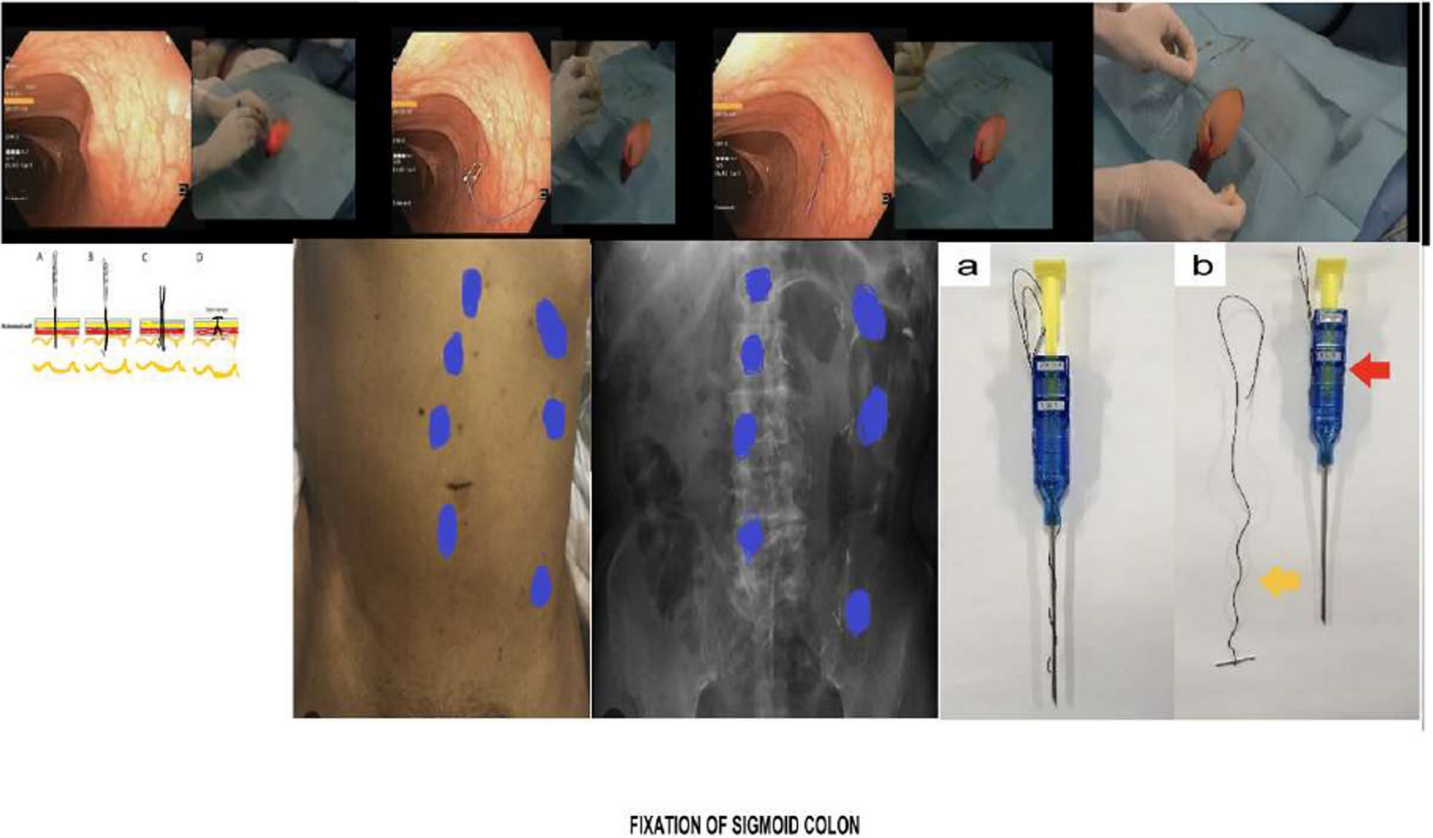
Percutaneous endoscopic sigmoid fixation technique. (Reference 12: Negm S., Farag A., Shafiq A., et al. Endoscopic management of acute sigmoid volvulus in high risk surgical elderly patients: a randomized controlled trial. Langenbecks Arch Surg 408,338(2023))
b *Surgical control group*: De-torsion mirrored the endoscopic approach. On postoperative day 2 or 3, patients underwent fixation or resection under general anesthesia, based on general condition and whether open or laparoscopic approach was used. Fixation employed interrupted transmural sutures to the anterior wall; sigmoidectomy was reserved for markedly redundant colons with elongated mesentery.
c *Follow-up*: Inpatient monitoring included daily clinical and laboratory evaluations, with contrast-enhanced computed tomography for any suspected leaks. Post-discharge surveillance extended at least 3 months, including clinic visits, colonoscopy, and imaging as clinically deemed necessary. Primary outcome: recurrence of volvulus inside 3 months (proven clinically or on imaging). Secondary: death at 30 and 90 days; any complications; length of hospital stay; time taken for the procedure; quality of life score at 6 months using WHOQOL-BREF; any late recurrences during available follow-up (median around 9 months).
d *Statistical analysis*: Data analysis utilized IBM SPSS Statistics version 23. Continuous variables were summarized as means ± standard deviations or medians (interquartile ranges), with ranges where appropriate; normality was assessed via Shapiro-Wilk testing. Group comparisons employed independent t-tests for parametric data or Mann-Whitney U tests for non-parametric distributions (standard deviation exceeding 30% of the mean). Categorical variables were expressed as frequencies and percentages, analyzed with chi-square or Fisher’s exact tests as needed. Statistical significance was set at *p* ≤ 0.05.

All patients initially underwent attempted detorsion via rectal tube or endoscopy, with definitive fixation deferred to postoperative day 2 or 3 to allow stabilization and colonic decompression.

a *Percutaneous Endoscopic Group*: Following triage to intensive care or ward based on clinical stability, patients received fluid resuscitation and supportive care. Detorsion proceeded via rectal tube or sedated colonoscopy (Olympus CF-260; Olympus, Tokyo, Japan) in the operating theater, with fluoroscopic guidance (C-arm) facilitating targeted insufflation and suction until resolution. A rectal tube was retained for ongoing decompression and irrigation (500 mL Ringer’s lactate every 8 h for 1–2 days) to clear fecal residue and prepare the colon, and patients remained nil per os during this phase.
  On postoperative day 2 or 3, percutaneous fixation was performed (Fig. 1):
  a Intravenous ceftriaxone and metronidazole were administered prophylactically prior to sedation.
  b Two surgeons collaborated: one performing colonoscopy, and the other deploying anchors for sigmoid colon fixation.
  c Patients were positioned in lithotomy, and radiopaque contrast was instilled endoscopically to locate the proposed sites of sigmoid colon fixation on the anterior abdominal wall.
  d Fixation sites were identified in the left lower quadrant via the guidance of endoscopic transillumination and external digital palpation then an artery forceps to replace the external digital palpation. Anchor placement followed patient-specific patterns: circular, parallel lines, or longitudinal along the sigmoid axis with 1 cm intervals, determined by abdominal contour, colonic position, and surgeon’s preference.
  e After marking any point of fixation, 2–3 mm skin incision was done using scalpel, the two-shot anchor device was passed through the incision into the sigmoid colon, before anchor device firing (the following step), we used a 20-gauge needle for further confirmation of site of fixation. Suction test was utilized via the 20-gauge needle to be sure that there was no any intervening organs between the sigmoid colon and anterior abdominal wall.
  f Devices included the Olympus two-shot anchor system (or alternatives such as Facial Closure Forceps or Endo Close, per surgeon’s experience and preference). The two-shot anchor device used only for fixation at one point, so multiple devices were used up to 4–5 devices as each one have two threads with T-shaped metal bar and two buttons with a double, click top button: (1) first thread with metal bar is released by clicking the first marked button followed by clicking the top top button for complete separation of the thread from the device; (2) second thread is released by clicking the second marked button followed by clicking the top button for complete separation from the device. Then the two threads are pulled up against anterior abdominal wall then are tied together.
  g After the first firing of the two-shot anchor device and releasing the first T-shape metal bar, the same device was introduced again through the same incision to release the second T-shaped metal bar, but after confirmation again that there was not any intervening organs between the sigmoid colon and anterior abdominal wall using a 20-gauge needle suction test.
  h The above steps were repeated again at multiple points of fixation (4–5 points). “Choosing the points of fixation relied on many factors such as sigmoid colon contour, its course and its accessibility, surgeon’s experience and the abdominal wall.”
  i Upon discharge, virtual colonoscopy was performed to confirme fixation integrity and exclude anastomotic leaks.

## Results

Of 70 screened patients, 4 withdrew post-detorsion, leaving 66 for analysis (33 per group). Males comprised 18 (27%), with 10 (30%) in the endoscopic group and 8 (24%) in the surgical group; females were 48 (73%), constituted 23 (70%) and 25 (76%) of the patients, respectively. Median age was 63 years overall (endoscopic 54 years vs. surgical 58 years). The median body mass index ranged 18–35 kg/m^2^ in both arms (Table 1).

**Table 1:** Baseline demographics, comorbidities, post-intervention complications, and quality-of-life outcomes

	All	Endoscopy group n = 33	Surgical control group n = 44	*P* value	statistical test
Sex	N (%)	N (%)	N (%)		
Male	18(27.3)	10(30.3)	8(24.2)	0.58	Chi square
Female	48(72.7)	23(69.7)	25(75.8)		
*Age (Years)*					
Median(IQR)	63(44–73)	54(45–64)	58(44–70)	0.133	Mann Whitney U
Range	30–80	30–80	30–80		
*Body mass index*					
Mean ± SD	27.7 ± 4.4	27.9 ± 4.3	27.5 ± 4.6	0.761	Independent t
Range	18–35	20–35	18–35		
*ASA (American Society of Anaesthesia)*					
ASA I	10(15.2)	8(24.2)	2(6.1)	**0.039**	Chi square
ASAII	10(15.2)	1(3)	9(27.3)	**0.006**	chi square
ASAIII	27(40.9)	10(30.3)	17(51.5)	0.08	Chi square
ASA IV	19(28.8)	14(42.4)	5(15.2)	**0.014**	chi square
*Comorbidities*					
Hypertension	19(28.8)	16(48.5)	3(9.1)	** < 0.001**	Chi square
Diabetes	23(34.8)	16(48.5)	7(21.2)	**0.02**	chi square
Ischemic heart disease	19(28.8)	14(42.4)	5(15.2)	**0.014**	Fisher Exact
Chronic liver disease	19(28.8)	14(42.4)	5(15.2)	**0.014**	Fisher Exact
Chronic renal failure	4(6.1)	2(6.1)	2(6.1)	1	Fisher Exact
Mild obesity	26(39.4)	13(39.4)	13(39.4)	1	Fisher Exact
Need for general anaesthesia	33(50)	0(0)	33(100)	< 0.001?	Fisher Exact
Post intervention complications					Fisher Exact
Small intestinal injury	3(4.5)	1(3)	2(6.1)	1	Fisher Exact
Sigmoid colon perforation	1(1.5)	1(3)	0(0)	1	Fisher Exact
Skin necrosis	6(9.1)	2(6.1)	4(12.1)	0.672	Fisher Exact
Chest infection	8(12.1)	3(9.1)	5(15.2)	0.708	Fisher Exact
peritoneal abscess	7(10.6)	3(9.1)	4(12.1)	1	Chi square
Peritonitis	7(10.6)	3(9.1)	4(12.1)	1	chi square
Faecal fistula	4(6.1)	0(0)	4(12.1)	0.114	Fisher Exact
Recurrence	2(3)	1(3)	1(3)	1	Chi square
Mortality	3(4.5)	0(0)	3(9.1)	0.238	chi square
*Quality of life*					
Excellent	41(62.1)	24(72.7)	17(51.5)	0.076	Chi square
Good	16(24.2)	5(15.2)	11(33.3)	0.085	chi square
Poor	9(13.6)	4(12.1)	5(15.2)	1	Fisher Exact

*P* value significant at ≤ 0.05

ASA classifications in the endoscopic group were I (n = 8, 24%), II (n = 1, 3%), III (n = 10, 30%), and IV (n = 14, 42%); surgical: I (n = 2, 6%), II (n = 9, 27%), III (n = 17, 51%), IV (n = 5, 15%). Comorbidities favored the endoscopic group for higher burdens: hypertension and diabetes each in 16 (49%), ischemic heart disease and chronic liver disease each in 14 (42%), renal failure in 2 (6%), and mild obesity in 13 (39%) patients, respectively; while in the surgical group, they were lower (3 [9%], 7 [21%], 5 [15.2%], 5 [15.2%], 2 [6.1%], 13 [39.4%], respectively). All surgical patients required general anesthesia (Table 1).

Procedure durations ranged 15–40 min endoscopically versus 60–120 min surgically (median 30 [IQR 25–35] vs. 100 [90–110] minutes, respectively, *p* < 0.001). Hospital stays were 1 day (IQR 1–2) versus 7 days (6–8, p < 0.001), respectively, while post-procedure observation periods were (1 [1, 2] vs. 7 [6–8] days, respectively, *p* < 0.001) (Table 2).

**Table 2:** Procedure duration, hospital length of stay, and post-procedure observation period

	All	Endoscopy group N = 33	Surgical control group N = 33	*P* value
*Intervention time in minutes*				
Median(IQR)	50(30–100)	30(25–35)	100(90–110)	< 0.001
Range	15–120	15–40	60–120	
*Hospital stay in days*				
Median(IQR)	3.5(1–7)	1(1–2)	7(6–8)	< 0.001
Range	1–15	1–2	5–15	
*Notice period after definitive management in days*				
Median(IQR)	3.5(1–7)	1(1–2)	7(6–8)	< 0.001
Range	1–15	1–2	5–15	

Statistical significance was assessed using the Mann–Whitney U test (*p* ≤ 0.05)

Post-procedure complications (Table 1) included small bowel injury (endoscopic 1 [3%] vs. surgical 2 [6%]), sigmoid perforation (1 [3%] vs. 0), skin necrosis (2 [6%] vs. 4 [12%]), pulmonary infection (3 [9%] vs. 5 [15%]), intra-abdominal abscess (3 [9%] vs. 4 [12%]), peritonitis (3 [9%] vs. 4 [12%]), fecal fistula (0 vs. 4 [12%]), recurrence (1 [3%] vs. 1 [3%]), and mortality (0 vs. 3 [9%]).

Quality-of-life evaluation at 6 months using the WHOQOL-BREF yielded excellent score in 24 (73%) endoscopically versus 17 (52%) surgically patients, good in 5 (15%) versus 11 (33%), and poor in 4 (12%) versus 5 (15%) (Table 1).

## Discussion

Optimal management of recurrent sigmoid volvulus following initial detorsion remains contentious, prompting our focus on adjunctive percutaneous fixation to the anterior abdominal wall via endoscopic anchors. Endoscopy’s intrinsic diagnostic-therapeutic modalities- mucosal inspection, torsion localization, and obstruction etiology- solidifies its role. Key advantages include shorter durations of the procedure, and the hospital stays, smooth recovery, better cosmesis, and avoidance of general anesthesia.

Fixation feasibility correlates inversely with adiposity; thinner habitus facilitates precise transabdominal anchoring, justifying our BMI threshold of 35 kg/m^2^. Sedation-only protocols rendered the approach feasible for ASA IV patients, avoiding the general anesthesia’s hazards.

Our findings align closely with Imakita et al., who reported on eight ASA > III patients (median age 72.5 years, ischemia excluded) undergoing colonoscopy-guided percutaneous sigmoidopexy: median procedure time was 72.5 min (fixation 16 min), isolated subcutaneous emphysema in one, and no peritonitis, bowel injury, collections, infections, or obstructions; zero recurrences over 1 year, with three unrelated deaths from aspiration [1]. Similarly, our endoscopic group demonstrated minimal recurrences (one potential under-fixation) and procedure related complications.

Endoscopic complications were limited: a single 20-gauge needle-induced small bowel injury and sigmoid perforation, both managed conservatively; two patients demonstrated skin necrosis from luminal leakage and were managed with local debridement; three pulmonary infections secondary to peritonitis; three abscess-peritonitis complexes from perforations and completed resolved via percutaneous drainage and antibiotics; and one recurrence, possibly from inadequate points of adhesion. The peritonitis and abscess episodes were contained and cleared up with antibiotics plus image-guided drains. No patients needed reoperation or laparotomy.

The complications in the surgical group reflected greater invasiveness: two small bowel injuries from adhesiolysis, four skin necrosis and pulmonary infections each from fistulas, four abscess-peritonitis complexes, four fecal fistulas (maybe related to suboptimal preparation or ischemia), and three deaths from sepsis-induced multi-organ failure. “A number of surgical complications appeared related to the greater tissue handling and technical steps needed for open/laparoscopic work in frail patients. Endoscopic problems were mostly limited to the fixation technique itself and were managed conservatively or with simple drainage, which fits with the overall less invasive approach.”

Quality of life, gauged via the WHOQOL-BREF (26 items across physical, psychological, social, and environmental domains, scored 0–100: < 50 poor, 50–75 good, > 75 excellent), favored the endoscopic group at 6 months that was attributable to smooth recovery, reduced inpatient hospital stay, avoidance of general anesthesia, and incision-free healing. “Resection with colostomy prevents recurrence on the long-term but frequently reduces quality of life in frail elderly through stoma complications, prolonged recovery, and loss of independence. Percutaneous fixation overcame those downsides and gave better WHOQOL-BREF scores at 6 months. When complications did happen in the endoscopic arm, they were low-grade, managed without escalating to surgery, and carried no mortality suggesting these very sick patients tolerated the approach better than a open/laparoscopic surgery.”

Follow-up reached a median of approximately 9 months (up to 14 months for the first enrolled patients), and no further volvulus episodes occurred beyond the single recurrence already noted at 3 months in each arm. Other published series on percutaneous endoscopic sigmoid fixation report no recurrences at 1–3 years in small high-risk groups (for instance Imakita 2019 followed to 25.5 months median with zero relapses). We continue to monitor the cohort for longer-term durability.”

Study limitations include modest sample size, exclusion of obese patients as obese patient have difficulty to identify points of sigmoid colon fixation due to excess fat, and operator-dependent technique variability (different tools used in sigmoid colon fixation like anchor device, port closure device or facial closure device). Strengths lie in its randomized design and real-world applicability.

## Conclusion

Percutaneous endoscopic fixation provides a lower-morbidity and mortality alternative approach for patients with recurrent sigmoid volvulus without the need for general anesthesia.

## Data Availability

All study data are contained within the manuscript and supplementary files; additional details are available from the corresponding author upon reasonable request.
